# m^6^A RNA Methylation Regulators Elicit Malignant Progression and Predict Clinical Outcome in Hepatocellular Carcinoma

**DOI:** 10.1155/2021/8859590

**Published:** 2021-06-04

**Authors:** Wenli Li, Jun Liu, Zhanzhong Ma, Xiaofeng Zhai, Binbin Cheng, Hetong Zhao

**Affiliations:** ^1^Reproductive Medicine Center, Yue Bei People's Hospital, Shaoguan, Guangdong, China; ^2^Department of Clinical Laboratory, Yue Bei People's Hospital, Shaoguan, Guangdong, China; ^3^Department of Traditional Chinese Medicine, Changhai Hospital, Naval Military Medical University, Shanghai, China

## Abstract

Hepatocellular carcinoma (HCC) is a leading cause of cancer-related death worldwide, and N6-methyladenosine (m^6^A) is a predominant internal modification of RNA in various cancers. We obtained the expression profiles of m^6^A-related genes for HCC patients from the International Cancer Genome Consortium and The Cancer Genome Atlas datasets. Most of the m^6^A RNA methylation regulators were confirmed to be differentially expressed among groups stratified by clinical characteristics and tissues. The clinical factors (including stage, grade, and gender) were correlated with the two subgroups (cluster 1/2). We identified an m^6^A RNA methylation regulator-based signature (including METTL3, YTHDC2, and YTHDF2) that could effectively stratify a high-risk subset of these patients by univariate and LASSO Cox regression, and receiver operating characteristic (ROC) analysis indicated that the signature had a powerful predictive ability. Immune cell analysis revealed that the genes in the signature were correlated with B cell, CD4 T cell, CD8 T cell, dendritic cell, macrophage, and neutrophil. Functional enrichment analysis suggested that these three genes may be involved in genetic and epigenetic events with known links to HCC. Moreover, the nomogram was established based on the signature integrated with clinicopathological features. The calibration curve and the area under ROC also demonstrated the good performance of the nomogram in predicting 3- and 5-year OS in the ICGC and TCGA cohorts. In summary, we demonstrated the vital role of m^6^A RNA methylation regulators in the initial presentation and progression of HCC and constructed a nomogram which would predict the clinical outcome and provide a basis for individualized therapy.

## 1. Introduction

Hepatocellular carcinoma (HCC), one of the most common malignancies, ranks second among the leading causes of cancer-related death globally [1]. The prognosis of HCC patients is relatively poor, mainly accompanied by liver cirrhosis or diagnosed at a late stage. Although the therapies of HCC have undergone rapid progress during the past decades, ranging from surgical and local treatment to molecular-targeted therapy and immunotherapy, the prognosis is undesirable [2, 3]. Therefore, it is imperative to clarify the molecular mechanisms of HCC to discover novel therapeutic targets and improve the treatment options.

N6-Methyladenosine (m^6^A), a predominant internal modification of RNA in mammalian cells, has been recognized as having a vital role in mRNA stability, export, translation, splicing, and decay [4]. The modification of m^6^A is conducted by three kinds of proteins: methyltransferases (called “writers”), m^6^A-binding proteins (called “readers”), and demethylases (called “erasers”) [5]. Moreover, m^6^A-dependent mRNA regulation is fundamental in different key biological processes, including embryonic development, stem cell differentiation [6], neurogenesis [5, 7], and stress responses [8].

Up to now, the implication of m^6^A has been studied in various cancers, such as glioblastoma [7], acute myeloid leukemia [9], breast cancer [10], and hepatocellular carcinoma [11]. However, little is known about its roles in initial presentation, development, and pathogenesis for HCC. Recently, bioinformatics research revealed that m^6^A-related genes including METTL3 and YTHDF1 were biological markers and independent prognosis factors in HCC [12]. Methyltransferase-like 14 (METTL14) was shown to be a prognosis factor for HCC and inhibited by microRNA 126 in HCC metastasis [13]. Methyltransferase-like 3 (METTL3) correlates with the poor prognosis of HCC and promotes the progression of HCC [11]. Wilms tumor 1-associated protein (WTAP) was investigated to be a poor prognosis factor and contributed to the progression of HCC via the HuR-ETS1-p21/p27 axis [14]. However, the biological functions' clinical value of other m^6^A-related genes in HCC remains unclear.

In this section, we comprehensively analyzed the expression levels of fourteen m^6^A RNA methylation regulators and clinical factors in patients with HCC from the ICGC (International Cancer Genome Consortium, https://icgc.org/), Gene Expression Omnibus (GEO, https://www.ncbi.nlm.nih.gov/geo/), and TCGA (The Cancer Genome Atlas, http://cancergenome.nih.gov/) databases. We uncovered the invaluable role of m^6^A RNA methylation regulators in the development of HCC and constructed a signature and a nomogram for predicting the survival of HCC.

## 2. Materials and Methods

### 2.1. Data Collection

The profiles were downloaded for 232 patients with HCC from ICGC-LIRI-JP, 209 patients with HCC from GEO-GSE14520, and 370 patients with HCC from TCGA-LIHC (Table 1) in August 2019. And the accession ID from TCGA and ICGC database is shown in Supplement Table 1. Patients who have insufficient clinicopathological data or “0” gene expression values were not included. Since the data come from TCGA and ICGC, it is not necessary to get the study approval by the ethics committee. The patients from the ICGC dataset were defined as a training cohort, and the patients from TCGA dataset were defined as a validation cohort. All statistical analyses were performed using R statistical software (version 3.6).

### 2.2. m^6^A RNA Methylation Regulator Selection

The m^6^A RNA methylation regulators were collected from published articles. Then, we selected the m^6^A RNA methylation regulators which were conformity to the genes from ICGC and TCGA. Next, these m^6^A RNA methylation regulators were further analyzed with clinical factors with HCC.

### 2.3. The Functional Enrichment Analysis

Gene Ontology (GO) analysis and Kyoto Encyclopedia of Genes and Genomes (KEGG) pathway categories were used for functional enrichment analysis. GO term and KEGG pathways with a *P* value under 0.1 were considered indicative of a statistically significant difference.

### 2.4. Consensus Clustering

Consensus clustering was performed using the ConsensusClusterPlus package in R to determine subgroups of HCC based on the m^6^A RNA methylation regulators [15, 16]. The cumulative distribution function (CDF) plots show consensus clustering for each *k* to find the appropriate *k* which reaches maximum stability.

### 2.5. Establishment of the Prognostic Signature

At first, we conducted a univariate Cox regression analysis to select prognostic genes. Next, LASSO (least absolute shrinkage and selection operator) Cox regression analysis was performed to choose independent high-risk genes for OS. Then, we built a prognostic signature derived from the multivariate Cox regression analysis including significant variables. The signature with the smallest Akaike information criterions (AICs) was selected and assessed by using Harrell's concordance index (C-index). Patients were divided into a high-risk group and a low-risk group based on the median score as the cut-off value. The receiver operating characteristic (ROC) curve and the area under ROC (AUC) were used to evaluate the calibration and discrimination of the signature for OS by the R package “survivalROC.” The Kaplan-Meier curve and log-rank test were drawn to analyze survival data by the R package “survival.”

### 2.6. Immune-Related Analysis

We used the tumor immune estimation resource (TIMER) database (http://cistrome.org/TIMER/) to analyze and visualize the abundances of tumor-infiltrating immune cells, such as B cells, CD4 T cells, CD8 T cells, macrophages, neutrophils, and dendritic cells.

### 2.7. Nomogram Establishment

The nomogram was built for prediction of 3- and 5-year survival based on the prognostic signature and clinical factors by the R package “rms.” The predictive value of the nomogram was evaluated using the calibration plot and ROC curve by the R packages “rms” and “timeROC.”

## 3. Results

### 3.1. The Relationship between m^6^A RNA Methylation Regulators and Clinical Factors

**Figure 1:**
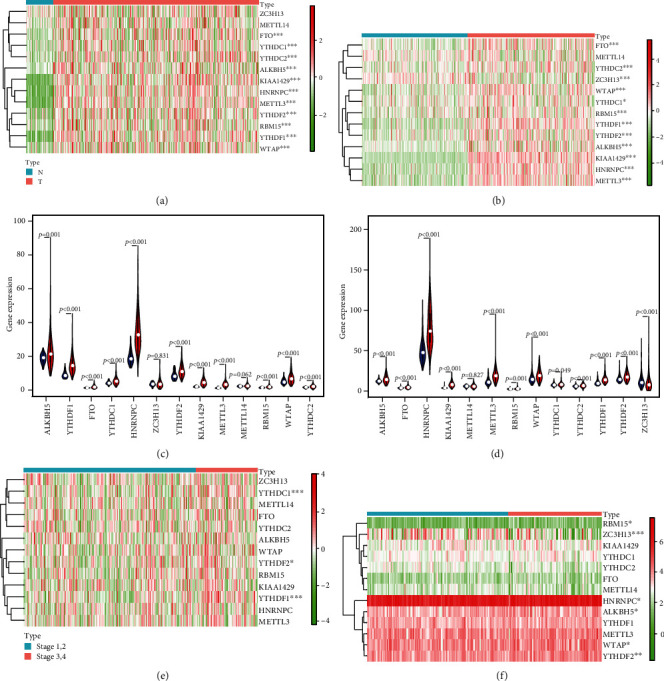
The expression levels of m^6^A RNA methylation regulators were associated with clinical factors. (a, b) The heatmaps for the expression levels of m^6^A RNA methylation regulators in HCC tumor and normal tissues in TCGA (a) and ICCA (b) cohorts. (c, d) The quantitative analyses for the expression levels of m^6^A RNA methylation regulators in HCC tumor and normal tissues in TCGA (c) and ICCA (d) cohorts. (e, f) The heatmaps for the expression levels of m^6^A RNA methylation regulators in stages 1 and 2 and stages 3 and 4 in TCGA (e) and ICCA (f) cohorts. ^∗^*P* < 0.05, ^∗∗^*P* < 0.01, ^∗∗∗^*P* < 0.001, and ^∗∗∗∗^*P* < 0.0001.

The fourteen m^6^A RNA methylation regulators collected from published literature evaluated the relationship between normal and tumor tissues in TCGA and ICGC cohorts (Figures 1(a) and 1(b)). The results showed that the expression levels of most m^6^A RNA methylation regulators were significantly associated with normal and tumor tissues. The results of quantitative analyses confirmed that the expression levels of the fourteen m^6^A RNA methylation regulators in tumor tissues were significantly higher than the expression levels in normal tissues except ZC3H13 and METTL14 in TCGA cohort (Figure 1(c)), and the results in the ICGC cohort were consistent with TCGA cohort except METTL14 (Figure 1(d)). The relationship between stages and the expression levels of the fourteen m^6^A RNA methylation regulators were also analyzed, and the results showed that the expression levels in patients with stages 3 and 4 were higher than those in patients with stages 1 and 2 in TCGA and ICGC cohorts (Figures 1(e) and 1(f)).

### 3.2. Consensus Clustering Identified Two Subgroups in HCC

**Figure 2:**
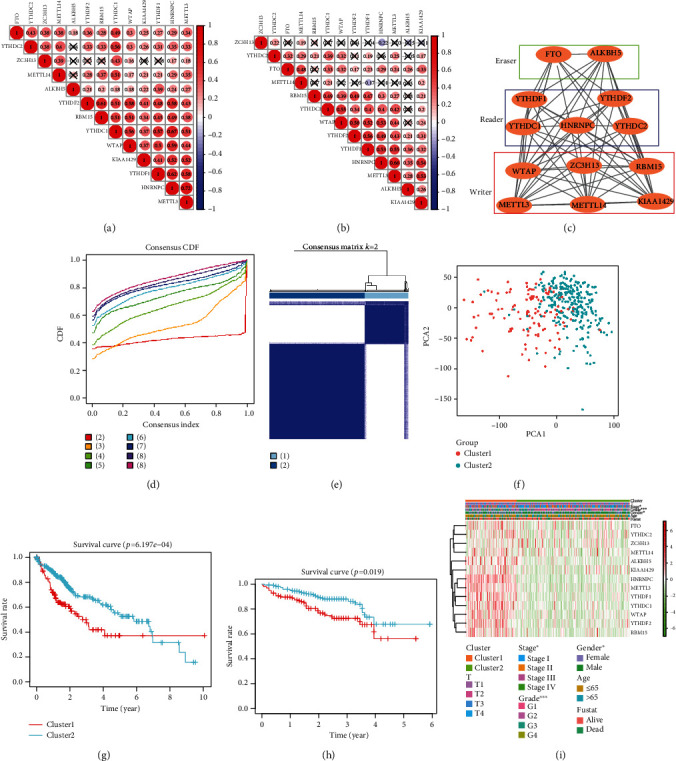
Identification of HCC subtypes using consensus clustering. (a, b) Spearman correlation analysis of the fourteen m^6^A modification regulators in the ICGC (a) and TCGA (b) cohorts. (c) The m^6^A modification-related interactions among the fourteen m^6^A RNA methylation regulators. (d) Consensus clustering CDF for *k* = 2 to *k* = 9. (e) Consensus clustering matrix of XXX TCGA samples for *k* = 2. (f) Principal component analysis of the total RNA expression profile in TCGA dataset. (g) Kaplan-Meier survival curves of clusters 1 and 2 in the ICGC cohort with *P* value. (h) Kaplan-Meier survival curves of clusters 1 and 2 in TCGA cohort with the *P* value. (i) In TCGA, the fustat represents the status of the patients.

To analyze the relationship among fourteen m^6^A RNA methylation regulators, the Spearman correlation analyses were used among fourteen m^6^A RNA methylation regulators in TCGA (Figure 2(a)) and ICGC cohorts (Figure 2(b)). The interaction of these proteins was retrieved from the STRING database (https://string-db.org/) (Figure 2(c)). To divide the patients with HCC based on consensus clustering of m^6^A RNA methylation regulators, we used a novel consensus clustering method to determine the prognostic capabilities in TCGA cohort. For each cluster number *k*, consensus clustering cumulative distribution function (CDF) of each final consensus matrix (FCM) was calculated (Figure 2(d)). As shown in Figures 2(e) and 2(f), we choose *k* = 2 to distinguish the patients with HCC more clearly. The survival analysis showed that cluster 1 patients had significantly poorer overall survival than cluster 2 patients in TCGA (*P* < 0.001) and ICGC (*P* < 0.05) cohorts. The clinical factors which included T stage, stage, grade, gender, age, and status were correlated with TCGA cohort (Figure 2(i)).

### 3.3. The Functional Enrichment Analysis

GO enrichment analysis of these regulators revealed that many of them were related to the GO terms “RNA modification,” “mRNA methylation,” “regulation of mRNA metabolic process,” and so on. KEGG analysis showed enrichment in several RNA-related pathways, including processing of capped intron-containing pre-mRNA and reversal of alkylation damage by DNA dioxygenases (Table 2).

### 3.4. The Prognostic Signature Based on the m^6^A RNA Methylation Regulators

**Figure 3:**
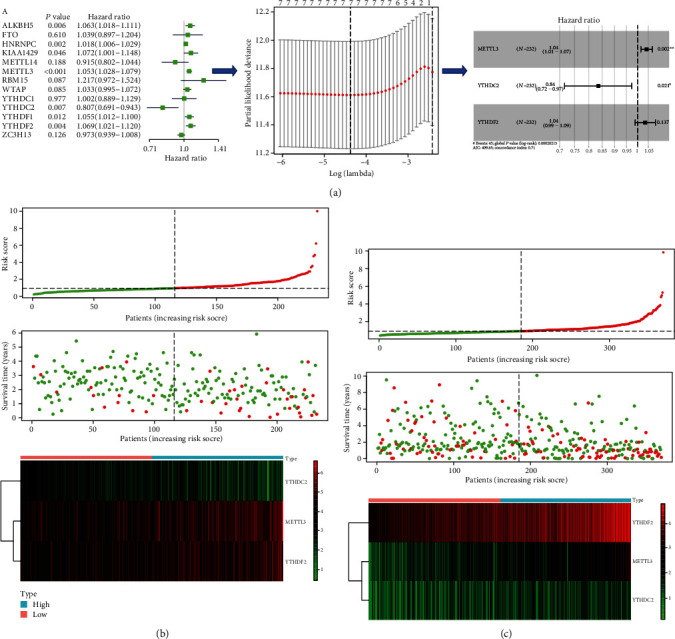
Construction of prognostic signature. (a) The procedure of the construction of the prognostic signature. (b, c) Correlation between the prognostic signature and the overall survival of patients in the ICGC cohort (b) and TCGA (c) cohorts. The distribution of risk scores (upper), survival time (middle), and gene expression levels (below). The black dotted lines represent the median risk score cut-off dividing patients into low- and high-risk groups. The red dots and lines represent the patients in high-risk groups. The green dots and lines represent the patients in low-risk groups.

To develop a prognostic signature, univariate Cox regression analysis and LASSO penalized Cox regression analysis were used to identify independent prognostic genes for OS in HCC (Figure 3(a)). The univariate and LASSO Cox regression analyses showed that ALKBH5, HNRNPC, KIAA1429, METTL3, YTHDC2, YTHDF1, and YTHDF2 were independent prognostic genes for OS in the ICGC cohort. Then, the multivariate Cox regression analysis identified three independent prognostic genes: METTL3, YTHDC2, and YTHDF2, and the risk score = (1.04 × the expression level of METTL3) + (−0.84 × the expression level of YTHDC2) + (1.04 × the expression level of YTHDF2). The C-index of the signature was up to 0.71, and the AIC was 409.65. The results represented that the signature had a reasonable ability to discriminate patients of poor prognosis from patients of favor prognosis. Each patient in the signature was calculated as a risk score. Using the median risk score value as the cut-off point, patients in each data portal were classified into low-risk and high-risk groups. We also figured the correlation between the prognostic signature and the overall survival of patients in the ICGC cohort (Figure 3(b)), TCGA (Figure 3(c)), and GSE14520 (Figure S1A) cohorts. The distribution of risk scores (upper), survival time (middle), and gene expression levels (below) are shown in Figures 3(b) and 3(c) and Supplement Figure 1A.

### 3.5. The Relationship between the Prognostic Signature and Clinical Factors

**Figure 4:**
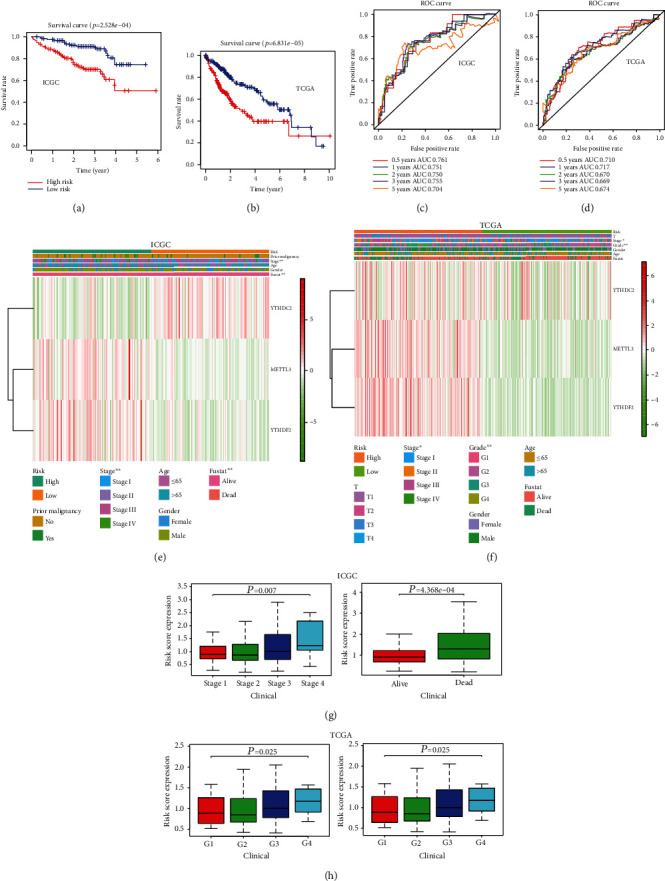
The differences between high- and low-risk groups in the ICGC and TCGA cohorts. (a, b) The Kaplan-Meier survival curves between high- and low-risk groups in the ICGC (a) and TCGA (b) cohorts. (c, d) ROC curves and AUC values in the ICGC (c) and TCGA (d) cohorts. (e, f) The relationship between the clinical factors and the risk groups based on the prognostic signature in the ICGC (e) and TCGA (f) cohorts. (g, h). The relationship between the clinical characteristics and the risk score based on the prognostic signature in the ICGC (g) and TCGA (h) cohorts. ^∗^*P* < 0.05 and ^∗∗^*P* < 0.01. The fustat represents the status of the patients.

The Kaplan-Meier curve, ROC, and AUC were used to assess the prognostic capacity of the prognostic signature. Patients in the high-risk group showed significantly poorer OS than patients in the low-risk group in the ICGC, TCGA, and GSE14520 cohorts (all *P* < 0.01; Figures 4(a) and 4(b) and Figure S1B). The AUCs for 0.5-, 1-, 2-, 3-, and 5-year OS were 0.761, 0.751, 0.750, 0.755, and 0.704; 0.710, 0.717, 0.670, 0.669, and 0.674; and 0.728, 0.631, 0.605, 0.622, and 0.631 for the ICGC, TCGA, and GSE14520 cohorts, respectively (Figures 4(c) and 4(d) and Figure S1C). The relationship between the risk score groups and clinical factors was further analyzed in the ICGC and TCGA cohorts (Figures 4(e) and 4(f)). It was confirmed that the differences between the high- and low-risk groups with regard to stage (*P* < 0.01) and status (*P* < 0.01) were significant in the ICGC cohort. The differences between the high- and low-risk groups with regard to stage (*P* < 0.05) and grade (*P* < 0.01) were significant in TCGA cohort. Moreover, we examined the relationship between the risk score groups, and immune cells were further analyzed in the ICGC and TCGA cohorts (Figures 4(g) and 4(h)). The results suggested that the differences between the high- and low-risk groups with regard to regulatory T cells (*P* < 0.001), naive B cells (*P* < 0.01), follicular helper T cells (*P* < 0.05), memory B cells (*P* < 0.01), and M0 macrophages (*P* < 0.01) were significant in the ICGC cohort. The results also suggested that the differences between the high- and low-risk groups with regard to CD8 T cells (*P* < 0.01), M0 macrophages (*P* < 0.001), and CD4 memory resting T cells (*P* < 0.01) were significant in TCGA cohort.

### 3.6. The Univariate and Multivariate Cox Regression Analyses of the Prognostic Signature

**Figure 5:**
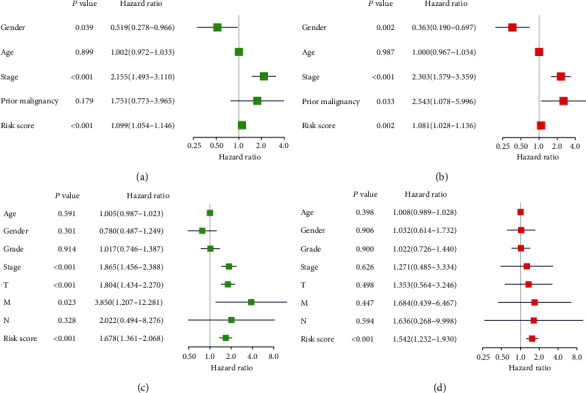
The univariate and multivariate Cox regression analyses of the prognostic signature. The univariate (a) and multivariate (b) Cox regression analyses of the prognostic signature in the ICGC cohort. The univariate (c) and multivariate (d) Cox regression analyses of the prognostic signature in TCGA cohort.

The univariate Cox regression analysis showed that stage and the risk score based on the signature were significant predictors of OS in the ICGC (stage: *P* < 0.001; risk score: *P* < 0.001; Figure 5(a)) and TCGA cohorts (stage: *P* < 0.001; risk score: *P* = 0.002; Figure 5(c)). The T and M stages were also related to OS (T stage: *P* < 0.001; M stage: *P* = 0.023; Figure 5(c)) in TCGA cohort. Moreover, multivariate Cox regression analysis confirmed that stage (hazard ratio (HR) = 2.303, 95% confidence interval (95% CI) 1.579–3.359; *P* < 0.001; Figure 5(b)) and the risk score based on the signature (HR = 1.081; 95% CI 1.028–1.136; *P* = 0.002; Figure 5(b)) were significant independent prognostic factors in the ICGC cohort. Multivariate Cox regression analysis further showed that the risk score based on the signature (HR = 1.542; 95% CI 1.232–1.930; *P* < 0.001; Figure 5(d)) was a significant independent prognostic factor in TCGA cohort. These data indicated that the risk score based on the signature was an independent predictor of HCC.

### 3.7. Nomogram Construction

**Figure 6:**
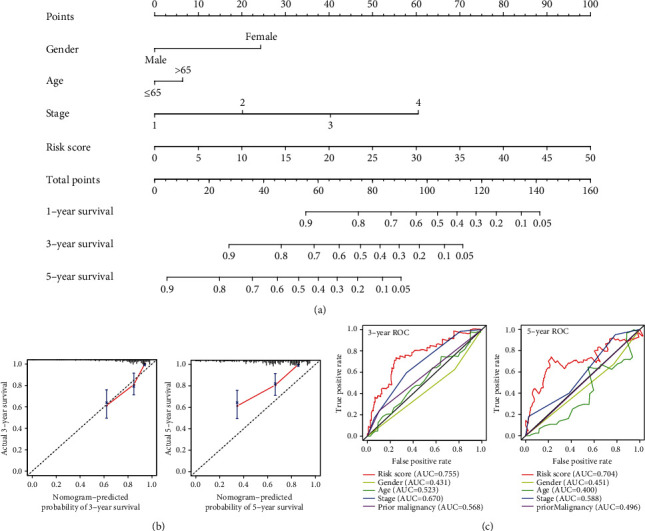
Nomogram, C-index, and ROC curves for predicting 3- and 5-year overall survival of patients with HCC in the ICGC cohort. (a) Nomogram based on the prognostic signature and clinical factors. (b) Calibration curve of the nomogram for predicting 3- and 5-year overall survival. (c) Comparison of the AUC value for predicting 3- and 5-year overall survival between the nomogram and clinical factors.

Based on the prognostic signature and clinical factors, such as gender, age, and stage, a nomogram was constructed (Figure 6(a)). The calibration curve was used to describe the prediction value of the nomogram, and the 45-degree line indicated the actual survival outcomes. The results for predicting 3- and 5-year OS indicated that the nomogram-predicted survival closely corresponded with the best prediction performance (Figure 6(b)). The 3-year AUC was 0.755 for nomogram, 0.431 for gender, 0.523 for age, 0.670 for stage, and 0.568 for prior malignancy. Moreover, the 5-year AUC was 0.704 for nomogram, 0.451 for gender, 0.400 for age, 0.588 for stage, and 0.496 for prior malignancy. These findings showed that compared with a single clinical factor, the nomogram combined with the signature and clinical factors had great predictive accuracy.

### 3.8. Immune Cell Analysis

**Figure 7:**
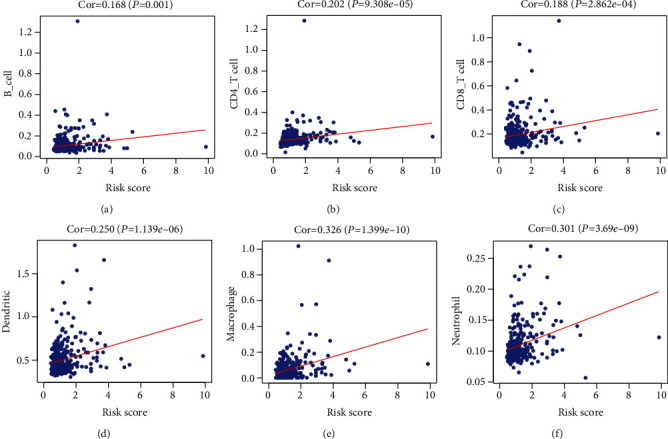
The Pearson correlation coefficient was calculated to determine the correlation among the B cell, CD4 T cell, CD8 T cell, dendritic cell, macrophage, and neutrophil cells.

The Pearson correlation analysis revealed that the risk score based on the signature in TCGA cohort was correlated with the B cell (*P* = 0.001), CD4 T cell (*P* < 0.001), CD8 T cell (*P* < 0.001), dendritic cell (*P* < 0.001), macrophage (*P* < 0.001), and neutrophil cells (*P* < 0.001) (Figure 7).

## 4. Discussion

Hepatocellular carcinoma (HCC) is a leading malignancy worldwide due to its high recurrence rate, high metastatic potential, and resistance to systematic therapy. However, the molecular mechanisms of HCC are unclear. The m^6^A RNA methylation is one of the most prevalent forms of RNA modifications. In recent decades, the high-throughput sequencing has revealed a significant role of m^6^A RNA methylation in HCC [17]. In the present study, we compared the expression levels of m^6^A RNA methylation regulators in tumor and normal tissues. The patients were divided into cluster 1 and cluster 2 according to consensus clustering. Based on the univariate Cox regression analysis and LASSO penalized Cox regression analysis, a prognostic signature was constructed with six m^6^A RNA methylation regulators and the signature was confirmed to be a significant independent prognostic. Next, the nomogram was developed with the prognostic signature and other clinical factors. Moreover, the nomogram also showed higher specificity and sensitivity for predicting 3- and 5-year survival for patients with HCC.

Until now, m^6^A RNA methylation regulators have attracted the attention of the medical research community. The fourteen m^6^A RNA methylation regulators from literature were analyzed using univariate and LASSO penalized Cox regression analysis in HCC. The results showed that METTL3, YTHDC2, and YTHDF2 were independent high-risk regulators in the ICGC and TCGA cohorts. The METTL3 and YTHDC2, also called “writer,” are core components of the m^6^A RNA methylation complex and involved in various biological processes. METTL3 was suggested to act as an oncogene in bladder cancer [18], breast cancer [19], ovarian carcinoma [20], and pancreatic cancer [21]. METTL3 has been also reported to be upregulated in HCC and associated with poor prognosis of HCC, and our findings confirmed the previous study that METTL3 plays an oncogenic role in HCC [11]. YTHDC2, the fifth member of the YTH protein family, was confirmed to be an oncogene in many cancers, and the expression level of YTHDC2 is high in several human cancer cells [22]. YTHDF2, termed “reader,” is a first studied functional m^6^A-binding protein that mainly regulates the stability of mRNA [23]. It acts as a tumor suppressor in HCC [24], acute myeloid leukemia [25], and pancreatic cancer [26]. These conclusions were consistent with the findings in our study. Here, we investigated that METTL3 and YTHDC2 were negatively correlated with the prognosis and YTHDF2 was positively associated with the prognosis.

Our study also established the prognostic signature and nomogram based on m^6^A RNA methylation regulators for predicting the outcome of patients with HCC. The genomic signature and nomogram, integrated multiple biomarkers, are promising methods that would improve clinical management. Recently, a risk signature, using seven m^6^A RNA methylation regulators, was built to predict the clinical outcomes of gliomas [27]. METTL3 and YTHDF1 were identified as independent poor prognostic factors in HCC [12]. Nevertheless, the clinical factors should be also considered to ameliorate clinical therapy. In our study, the nomogram was constructed and validated based on the prognostic signature and clinical factors. Compared with other factors, the nomogram showed a more robust ability to predict the 3- and 5-year OS.

There are still several limitations. At the beginning, the prognostic value of the signature was not yet confirmed by the validation experimental studies in HCC *in vitro* and *in vivo*, which was just validated by another online dataset. Second, the sample size of the training and validation cohorts is quite small. Further validations are awaited.

In all, we have performed the first signature based on the m^6^A RNA methylation regulator, as well as the construction of the nomogram based on the signature and clinical factors. Hence, this prognostic signature would be a useful marker for guiding the selection of individualizing therapy for HCC. Future studies should focus on the underlying molecular mechanisms.

## Figures and Tables

**Table 1:** Characteristics of patients with HCC in TCGA and ICGC cohorts.

Clinical characteristics		Total	%
TCGA		370	
Survival status	Survival	244	65.95
	Death	126	34.05
Age	≤65 years	232	62.70
	>65 years	138	37.30
Gender	Male	249	67.30
	Female	121	32.70
Histological grade	G1	55	14.86
	G2	177	47.84
	G3	121	32.70
	G4	12	3.24
Stage	I	171	46.22
	II	85	22.97
	III	85	22.97
	IV	5	1.35
T classification	T1	181	48.92
	T2	93	25.14
	T3	80	21.62
	T4	13	3.51
	TX	1	0.27
M classification	M0	266	71.89
	M1	4	1.08
	MX	100	27.03
N classification	N0	252	68.11
	N1	4	1.08
	NX	113	30.54

ICGC		232	

Survival status	Survival	189	81.47
	Death	43	18.53
Age	≤65 years	90	38.79
	>65 years	142	61.21
Gender	Male	171	73.71
	Female	61	26.29
Stage	I	36	15.52
	II	106	45.69
	III	71	30.60
	IV	19	8.19
Prior malignancy	No	202	87.07
	Yes	30	12.93

GSE14520		209	

Survival status	Survival	130	62.20
	Death	79	37.80

Abbreviations: TCGA: The Cancer Genome Atlas; ICGC: International Cancer Genome Consortium.

**Table 2:** Functional enrichment analysis for the prognostic signature.

Category	Term	Description	Count	FDR
GOTERM_BP	GO:0009451	RNA modification	8	5.42*E* − 12
GOTERM_BP	GO:0080009	mRNA methylation	5	1.45*E* − 10
GOTERM_BP	GO:1903311	Regulation of mRNA metabolic process	8	1.45*E* − 10
GOTERM_BP	GO:0006397	mRNA processing	9	1.71*E* − 10
GOTERM_BP	GO:0001510	RNA methylation	6	4.02*E* − 10
GOTERM_BP	GO:0016070	RNA metabolic process	11	8.34*E* − 06
GOTERM_BP	GO:0006396	RNA processing	10	4.02*E* − 10
GOTERM_MF	GO:1990247	N6-methyladenosine-containing RNA binding	5	3.23*E* − 12
GOTERM_MF	GO:0003723	RNA binding	8	5.30*E* − 07
GOTERM_MF	GO:0140098	Catalytic activity, acting on RNA	5	5.14*E* − 05
GOTERM_MF	GO:0016422	mRNA (2′-O-methyladenosine-N6-)-methyltransferase activity	2	7.72*E* − 05
GOTERM_MF	GO:0003729	mRNA binding	4	9.27*E* − 05
GOTERM_MF	GO:0035515	Oxidative RNA demethylase activity	2	9.27*E* − 05
GOTERM_CC	GO:0036396	RNA N6-methyladenosine methyltransferase complex	6	3.62*E* − 15
GOTERM_CC	GO:0016607	Nuclear speck	8	3.67*E* − 10
GOTERM_CC	GO:0005654	Nucleoplasm	10	3.39*E* − 05
GOTERM_CC	GO:1902494	Catalytic complex	7	4.16*E* − 05
Pathway	HSA-72203	Processing of capped intron-containing pre-mRNA	4	0.00017
Pathway	HSA-73943	Reversal of alkylation damage by DNA dioxygenases	2	0.00017

Abbreviations: GOTERM: Gene Ontology term; BP: biological process; MF: molecular function; CC: cellular component.

## Data Availability

All data generated or analyzed during this study are included in this published article.

## Abbreviations

HCC: Hepatocellular carcinoma
m^6^A: N6-Methyladenosine
ICGC: International Cancer Genome Consortium
TCGA: The Cancer Genome Atlas
ROC: Dependent receiver operating characteristic
LASSO: Least absolute shrinkage and selection operator
OS: Overall survival
CDF: Cumulative distribution function
GO: Gene Ontology
KEGG: Kyoto Encyclopedia of Genes and Genomes.

## Supplementary Materials

Supplementary 1 Figure S1: verification of prognostic signature in the GSE14520 cohort. (A) The distribution of risk scores (upper), survival time (middle), and gene expression levels (below). (B) The Kaplan-Meier survival curves between high- and low-risk groups. (C) ROC curves and AUC values of the signature.

Supplementary 2 Table S1: accession ID.
