# Supplementary material for: m6A RNA Methylation Regulators Elicit Malignant Progression and Predict Clinical Outcome in Hepatocellular Carcinoma
Source: Dis Markers. 2021 Jun 4;2021:8859590. doi: 10.1155/2021/8859590 (PMC8218914; doi:10.1155/2021/8859590)
Supplement: Supplementary 1 — Figure S1: verification of prognostic signature in the GSE14520 cohort. (A) The distribution of risk scores (upper), survival time (middle), and gene expression levels (below). (B) The Kaplan-Meier survival curves between high- and low-risk groups. (C) ROC curves and AUC values of the signature. [file 8859590.f1.docx]

Table S1. Accession id.

| TCGA-LIHC-ID | ICGC-LIRI-JP-ID |
| --- | --- |
| TCGA-EP-A12J-11A-11R-A131-07 | SP99273-DO45275-N |
| TCGA-BC-A10T-11A-11R-A131-07 | SP192715-DO50816-N |
| TCGA-G3-A3CH-11A-11R-A22L-07 | SP192687-DO48751-N |
| TCGA-BD-A3EP-11A-12R-A22L-07 | SP135318-DO45227-N |
| TCGA-BC-A10W-11A-11R-A131-07 | SP135231-DO45133-N |
| TCGA-FV-A23B-11A-11R-A16W-07 | SP192417-DO23515-N |
| TCGA-DD-A113-11A-12R-A131-07 | SP135229-DO45131-N |
| TCGA-DD-A3A2-11A-11R-A213-07 | SP192665-DO48715-N |
| TCGA-FV-A3R2-11A-11R-A22L-07 | SP192693-DO50783-N |
| TCGA-DD-A114-11A-12R-A131-07 | SP135249-DO45165-N |
| TCGA-BC-A10Z-11A-11R-A131-07 | SP135221-DO45113-N |
| TCGA-DD-A3A1-11A-11R-A213-07 | SP192674-DO48727-N |
| TCGA-EP-A3RK-11A-11R-A22L-07 | SP135314-DO45225-N |
| TCGA-DD-A11A-11A-11R-A131-07 | SP192448-DO48677-N |
| TCGA-EP-A26S-11A-12R-A16W-07 | SP99331-DO45303-N |
| TCGA-DD-A119-11A-11R-A131-07 | SP192717-DO50820-N |
| TCGA-DD-A116-11A-12R-A26B-07 | SP192690-DO48761-N |
| TCGA-BC-A10U-11A-11R-A131-07 | SP192640-DO48697-N |
| TCGA-FV-A2QR-11A-11R-A213-07 | SP135245-DO45161-N |
| TCGA-FV-A3I0-11A-11R-A22L-07 | SP135223-DO45119-N |
| TCGA-DD-A3A4-11A-11R-A22L-07 | SP135219-DO45111-N |
| TCGA-DD-A39Z-11A-21R-A213-07 | SP192636-DO48695-N |
| TCGA-DD-A11C-11A-11R-A131-07 | SP135344-DO45247-N |
| TCGA-DD-A1EH-11A-11R-A131-07 | SP192702-DO50802-N |
| TCGA-BC-A216-11A-11R-A155-07 | SP135206-DO45092-N |
| TCGA-DD-A3A6-11A-11R-A22L-07 | SP135208-DO45093-N |
| TCGA-DD-A1EL-11A-11R-A155-07 | SP192700-DO50799-N |
| TCGA-DD-A1EJ-11A-11R-A155-07 | SP192532-DO45235-N |
| TCGA-FV-A3I1-11A-11R-A22L-07 | SP192420-DO45175-N |
| TCGA-BC-A10R-11A-11R-A131-07 | SP192681-DO48737-N |
| TCGA-DD-A3A3-11A-11R-A22L-07 | SP192438-DO45191-N |
| TCGA-DD-A1EI-11A-11R-A131-07 | SP135338-DO45243-N |
| TCGA-BC-A10X-11A-11R-A131-07 | SP192263-DO23511-N |
| TCGA-BC-A110-11A-11R-A131-07 | SP192708-DO227801-N |
| TCGA-DD-A39W-11A-11R-A213-07 | SP192696-DO50789-N |
| TCGA-DD-A3A5-11A-11R-A22L-07 | SP135272-DO23517-N |
| TCGA-DD-A118-11A-11R-A131-07 | SP192261-DO227643-N |
| TCGA-BD-A2L6-11A-21R-A213-07 | SP192686-DO48747-N |
| TCGA-ES-A2HT-11A-11R-A180-07 | SP192679-DO48733-N |
| TCGA-DD-A1EG-11A-11R-A213-07 | SP135227-DO45125-N |
| TCGA-DD-A11D-11A-12R-A131-07 | SP191959-DO45139-N |
| TCGA-DD-A3A8-11A-11R-A22L-07 | SP135247-DO45163-N |
| TCGA-DD-A1EC-11A-11R-A131-07 | SP192680-DO48736-N |
| TCGA-BC-A10Q-11A-11R-A131-07 | SP192471-DO45211-N |
| TCGA-DD-A1EE-11A-11R-A131-07 | SP192649-DO45261-N |
| TCGA-BC-A10Y-11A-11R-A131-07 | SP192657-DO23552-N |
| TCGA-DD-A1EB-11A-11R-A131-07 | SP192537-DO23531-N |
| TCGA-DD-A11B-11A-11R-A131-07 | SP192725-DO50857-N |
| TCGA-DD-A39X-11A-11R-A213-07 | SP192457-DO48679-N |
| TCGA-DD-A39V-11A-11R-A213-07 | SP192697-DO50791-N |
| TCGA-CC-A3M9-01A-11R-A213-07 | SP192694-DO50785-N |
| TCGA-DD-A3A5-01A-11R-A22L-07 | SP192663-DO48706-N |
| TCGA-FV-A3I1-01A-11R-A22L-07 | SP191843-DO45107-N |
| TCGA-DD-AAEB-01A-11R-A41C-07 | SP192689-DO48759-N |
| TCGA-5C-AAPD-01A-21R-A39D-07 | SP135210-DO45094-N |
| TCGA-EP-A26S-01A-11R-A16W-07 | SP135350-DO45249-N |
| TCGA-BD-A2L6-01A-11R-A213-07 | SP192427-DO45183-N |
| TCGA-UB-A7ME-01A-11R-A33J-07 | SP192495-DO23525-N |
| TCGA-DD-A4NK-01A-11R-A28V-07 | SP192644-DO23550-N |
| TCGA-DD-AACS-01A-11R-A41C-07 | SP107009-DO48681-N |
| TCGA-DD-A39W-01A-11R-A213-07 | SP191879-DO45117-N |
| TCGA-DD-AACV-01A-11R-A41C-07 | SP192672-DO48723-N |
| TCGA-CC-A7IG-01A-11R-A33J-07 | SP192523-DO23529-N |
| TCGA-DD-AADV-01A-11R-A39D-07 | SP192498-DO23526-N |
| TCGA-CC-A8HS-01A-11R-A36F-07 | SP135361-DO45259-N |
| TCGA-DD-AADJ-01A-11R-A41C-07 | SP135333-DO45239-N |
| TCGA-DD-AACI-01A-11R-A41C-07 | SP192723-DO50850-N |
| TCGA-DD-AAD0-01A-11R-A41C-07 | SP192562-DO23537-N |
| TCGA-DD-A114-01A-11R-A131-07 | SP192703-DO50803-N |
| TCGA-2Y-A9H6-01A-11R-A39D-07 | SP135214-DO45099-N |
| TCGA-G3-A25U-01A-11R-A16W-07 | SP192556-DO23536-N |
| TCGA-FV-A3R2-01A-11R-A22L-07 | SP192669-DO48719-N |
| TCGA-ZS-A9CG-01A-11R-A37K-07 | SP192712-DO50809-N |
| TCGA-DD-A1EG-01A-11R-A213-07 | SP192670-DO48720-N |
| TCGA-RC-A7S9-01A-11R-A33R-07 | SP192699-DO50798-N |
| TCGA-CC-A8HT-01A-11R-A36F-07 | SP191958-DO45139-N |
| TCGA-2Y-A9H2-01A-12R-A38B-07 | SP192666-DO48716-N |
| TCGA-DD-AADC-01A-11R-A41C-07 | SP135365-DO45263-N |
| TCGA-EP-A2KA-01A-11R-A180-07 | SP135259-DO45173-N |
| TCGA-DD-AACA-01A-11R-A41C-07 | SP135253-DO45169-N |
| TCGA-MI-A75E-01A-11R-A32O-07 | SP192541-DO23532-N |
| TCGA-DD-A118-01A-11R-A131-07 | SP192673-DO48725-N |
| TCGA-BC-A10Q-01A-11R-A131-07 | SP192698-DO50793-N |
| TCGA-DD-AACA-02A-11R-A41C-07 | SP192688-DO48757-N |
| TCGA-BC-A3KG-01A-11R-A213-07 | SP192395-DO45153-N |
| TCGA-CC-5263-01A-01R-A131-07 | SP135243-DO45159-N |
| TCGA-BC-A69H-01A-11R-A311-07 | SP192671-DO48721-N |
| TCGA-DD-A11A-01A-11R-A131-07 | SP192661-DO45269-N |
| TCGA-DD-AADR-01A-11R-A41C-07 | SP135377-DO45281-N |
| TCGA-DD-A3A7-01A-11R-A22L-07 | SP135233-DO45137-N |
| TCGA-DD-A11C-01A-11R-A131-07 | SP192631-DO45257-N |
| TCGA-DD-AAD8-01A-11R-A41C-07 | SP192437-DO48674-N |
| TCGA-CC-A8HV-01A-11R-A36F-07 | SP192485-DO45215-N |
| TCGA-DD-AAVW-01A-11R-A41C-07 | SP135396-DO45305-N |
| TCGA-DD-AAW0-01A-11R-A41C-07 | SP50140-DO23530-N |
| TCGA-DD-AAC9-01A-11R-A41C-07 | SP135212-DO45097-N |
| TCGA-DD-AAC8-01A-11R-A41C-07 | SP191892-DO45121-N |
| TCGA-XR-A8TE-01A-11R-A36F-07 | SP135251-DO45167-N |
| TCGA-DD-AAVU-01A-11R-A41C-07 | SP192603-DO23542-N |
| TCGA-DD-AAVV-01A-11R-A41C-07 | SP135289-DO45213-N |
| TCGA-CC-5259-01A-31R-A213-07 | SP191868-DO45115-N |
| TCGA-G3-A7M7-01A-12R-A352-07 | SP99323-DO45299-N |
| TCGA-NI-A8LF-01A-11R-A36F-07 | SP135329-DO45237-N |
| TCGA-ED-A4XI-01A-11R-A266-07 | SP99319-DO45297-N |
| TCGA-BC-A5W4-01A-11R-A28V-07 | SP135384-DO45283-N |
| TCGA-DD-A4NF-01A-11R-A27V-07 | SP192721-DO50840-N |
| TCGA-DD-AACT-01A-11R-A41C-07 | SP192692-DO50778-N |
| TCGA-DD-AADK-01A-11R-A41C-07 | SP192507-DO23527-N |
| TCGA-BC-A8YO-01A-11R-A37K-07 | SP192709-DO50807-N |
| TCGA-4R-AA8I-01A-11R-A38B-07 | SP192545-DO48682-N |
| TCGA-FV-A23B-01A-11R-A16W-07 | SP192415-DO48672-N |
| TCGA-BC-A112-01A-11R-A131-07 | SP192397-DO45155-N |
| TCGA-ED-A459-01A-11R-A266-07 | SP135283-DO45203-N |
| TCGA-BD-A3EP-01A-11R-A22L-07 | SP191784-DO45096-N |
| TCGA-G3-A6UC-01A-21R-A33J-07 | SP192662-DO48704-N |
| TCGA-2Y-A9H9-01A-21R-A39D-07 | SP191925-DO23509-N |
| TCGA-DD-AAEK-01A-11R-A41C-07 | SP192442-DO23516-N |
| TCGA-DD-AAD5-01A-11R-A41C-07 | SP135256-DO45171-N |
| TCGA-G3-A5SK-01A-11R-A27V-07 | SP192664-DO48712-N |
| TCGA-DD-A3A2-01A-11R-A213-07 | SP192577-DO23538-N |
| TCGA-CC-5262-01A-01R-A131-07 | SP192695-DO50787-N |
| TCGA-DD-A4NP-01A-11R-A28V-07 | SP192434-DO45187-N |
| TCGA-DD-AAED-01A-12R-A41C-07 | SP191761-DO45095-N |
| TCGA-DD-A73E-01A-12R-A32O-07 | SP192651-DO45265-N |
| TCGA-CC-A5UD-01A-11R-A28V-07 | SP192411-DO23514-N |
| TCGA-DD-AACK-01A-11R-A41C-07 | SP135216-DO45101-N |
| TCGA-DD-A1EB-01A-11R-A131-07 | SP192548-DO23534-N |
| TCGA-ZP-A9CV-01A-11R-A38B-07 | SP192701-DO50800-N |
| TCGA-ED-A7XO-01A-11R-A352-07 | SP192654-DO45267-N |
| TCGA-2Y-A9GZ-01A-11R-A39D-07 | SP192713-DO50813-N |
| TCGA-ZP-A9D0-01A-11R-A37K-07 | SP192593-DO23541-N |
| TCGA-DD-AAEE-01A-11R-A41C-07 | SP191850-DO45109-N |
| TCGA-ZS-A9CD-01A-11R-A37K-07 | SP192605-DO23543-N |
| TCGA-CC-A8HU-01A-11R-A36F-07 | SP192705-DO50805-N |
| TCGA-GJ-A9DB-01A-11R-A37K-07 | SP192718-DO50822-N |
| TCGA-DD-AACA-02B-11R-A41C-07 | SP135295-DO45217-N |
| TCGA-G3-A25T-01A-11R-A16W-07 | SP192480-DO23524-N |
| TCGA-G3-A7M6-01A-11R-A33R-07 | SP135241-DO45145-N |
| TCGA-DD-A73C-01A-12R-A33J-07 | SP191982-DO23510-N |
| TCGA-KR-A7K8-01A-11R-A33J-07 | SP192682-DO48741-N |
| TCGA-2Y-A9HB-01A-11R-A39D-07 | SP192704-DO50804-N |
| TCGA-DD-AAVR-01A-11R-A41C-07 | SP192659-DO48701-N |
| TCGA-DD-AAVP-01A-11R-A41C-07 | SP135353-DO45251-N |
| TCGA-DD-A73A-01A-12R-A32O-07 | SP135394-DO45289-N |
| TCGA-2Y-A9GV-01A-11R-A38B-07 | SP192555-DO23535-N |
| TCGA-EP-A2KB-01A-11R-A180-07 | SP191956-DO45139-N |
| TCGA-WJ-A86L-01A-12R-A39D-07 | SP192691-DO50774-N |
| TCGA-2Y-A9GY-01A-11R-A38B-07 | SP192668-DO48717-N |
| TCGA-DD-AADM-01A-11R-A41C-07 | SP135300-DO45219-N |
| TCGA-DD-AACJ-01A-11R-A41C-07 | SP135392-DO45287-N |
| TCGA-CC-A7IE-01A-21R-A38B-07 | SP135279-DO45197-N |
| TCGA-GJ-A6C0-01A-12R-A311-07 | SP135203-DO45091-N |
| TCGA-DD-A1EJ-01A-11R-A155-07 | SP135235-DO45141-N |
| TCGA-XR-A8TF-01A-11R-A36F-07 | SP135358-DO45253-N |
| TCGA-2V-A95S-01A-11R-A37K-07 | SP192719-DO50825-N |
| TCGA-K7-AAU7-01A-11R-A38B-07 | SP192727-DO50859-N |
| TCGA-CC-A7IL-01A-11R-A33R-07 | SP191914-DO23508-N |
| TCGA-G3-AAV7-01A-11R-A38B-07 | SP192439-DO45193-N |
| TCGA-DD-A3A1-01A-11R-A213-07 | SP192619-DO23545-N |
| TCGA-DD-A11D-01A-11R-A131-07 | SP192678-DO48732-N |
| TCGA-CC-A9FU-01A-11R-A37K-07 | SP135341-DO45245-N |
| TCGA-DD-AAE6-01A-11R-A41C-07 | SP192454-DO45199-N |
| TCGA-DD-AADL-01A-11R-A41C-07 | SP135322-DO45231-N |
| TCGA-DD-AADS-01A-11R-A41C-07 | SP192405-DO23513-N |
| TCGA-CC-A7II-01A-11R-A33J-07 | SP192655-DO48700-N |
| TCGA-5R-AAAM-01A-12R-A41C-07 | SP135263-DO45189-N |
| TCGA-CC-A7IK-01A-12R-A33R-07 | SP135401-DO45307-N |
| TCGA-DD-AAE9-01A-11R-A41C-07 | SP192675-DO48728-N |
| TCGA-DD-AAD1-01A-11R-A41C-07 | SP192422-DO45179-N |
| TCGA-UB-A7MF-01A-11R-A33J-07 | SP192724-DO50855-N |
| TCGA-BC-A10Y-01A-11R-A131-07 | SP192683-DO48743-N |
| TCGA-FV-A495-01A-11R-A266-07 | SP192685-DO48746-N |
| TCGA-KR-A7K7-01A-11R-A33J-07 | SP192511-DO23528-N |
| TCGA-CC-5258-01A-01R-A131-07 | SP135269-DO45195-N |
| TCGA-5R-AA1D-01A-11R-A38B-07 | SP192627-DO23548-N |
| TCGA-ED-A97K-01A-21R-A38B-07 | SP135306-DO45221-N |
| TCGA-LG-A6GG-01A-11R-A311-07 | SP192543-DO23533-N |
| TCGA-DD-AADF-01A-11R-A41C-07 | SP192445-DO23518-N |
| TCGA-CC-A9FV-01A-11R-A37K-07 | SP192716-DO50818-N |
| TCGA-MI-A75G-01A-11R-A32O-07 | SP192711-DO50808-N |
| TCGA-DD-A39V-01A-11R-A213-07 | SP192425-DO45181-N |
| TCGA-DD-AACF-01A-11R-A41C-07 | SP135237-DO45143-N |
| TCGA-G3-AAV0-01A-11R-A37K-07 | SP192469-DO45209-N |
| TCGA-KR-A7K2-01A-12R-A33R-07 | SP192390-DO23512-N |
| TCGA-RC-A6M3-01A-11R-A32O-07 | SP192652-DO23551-N |
| TCGA-RC-A7SF-01A-11R-A352-07 | SP192419-DO45173-N |
| TCGA-DD-A39Y-01A-11R-A213-07 | SP192706-DO50806-N |
| TCGA-ES-A2HT-01A-12R-A180-07 | SP135309-DO45223-N |
| TCGA-DD-AADA-01A-11R-A41C-07 | SP191939-DO45135-N |
| TCGA-DD-A4NI-01A-11R-A27V-07 | SP192720-DO50832-N |
| TCGA-G3-A3CI-01A-11R-A213-07 | SP192676-DO48730-N |
| TCGA-WX-AA46-01A-11R-A39D-07 | SP135261-DO45177-N |
| TCGA-DD-AACD-01A-11R-A41C-07 | SP135368-DO45277-N |
| TCGA-DD-A73G-01A-22R-A32O-07 | SP135370-DO45279-N |
| TCGA-2Y-A9H0-01A-11R-A38B-07 | SP192393-DO45149-N |
| TCGA-DD-AACC-01A-11R-A41C-07 | SP135385-DO45285-N |
| TCGA-DD-A1EE-01A-11R-A131-07 | SP135218-DO45103-N |
| TCGA-FV-A4ZQ-01A-11R-A266-07 | SP192477-DO23523-N |
| TCGA-G3-AAV3-01A-11R-A37K-07 | SP192464-DO23519-N |
| TCGA-BC-4073-01B-02R-A131-07 | SP191903-DO45127-N |
| TCGA-G3-A3CH-01A-11R-A22L-07 | SP191920-DO45129-N |
| TCGA-G3-A3CK-01A-11R-A213-07 | SP135225-DO45123-N |
| TCGA-DD-A116-01A-11R-A131-07 | SP99137-DO45207-T |
| TCGA-5R-AA1C-01A-11R-A41C-07 | SP99213-DO45245-T |
| TCGA-DD-AAE4-01A-11R-A41C-07 | SP50153-DO23536-T |
| TCGA-DD-AACL-01A-11R-A41C-07 | SP50139-DO23529-T |
| TCGA-DD-AADW-01A-11R-A39D-07 | SP99325-DO45299-T |
| TCGA-DD-AACU-01A-11R-A41C-07 | SP98913-DO45097-T |
| TCGA-G3-A7M5-01A-11R-A33R-07 | SP50149-DO23534-T |
| TCGA-BW-A5NP-01A-11R-A27V-07 | SP99053-DO45165-T |
| TCGA-G3-A5SJ-01A-11R-A27V-07 | SP112184-DO45139-T |
| TCGA-DD-AACN-01A-11R-A41C-07 | SP50109-DO23514-T |
| TCGA-O8-A75V-01A-11R-A32O-07 | SP99077-DO45177-T |
| TCGA-ED-A5KG-01A-11R-A27V-07 | SP98985-DO45133-T |
| TCGA-G3-AAV5-01A-11R-A37K-07 | SP99189-DO45233-T |
| TCGA-ZP-A9CY-01A-11R-A38B-07 | SP99113-DO45195-T |
| TCGA-DD-A73B-01A-12R-A32O-07 | SP99065-DO45171-T |
| TCGA-MR-A520-01A-11R-A266-07 | SP107104-DO48733-T |
| TCGA-CC-A3MC-01A-11R-A22L-07 | SP99275-DO45275-T |
| TCGA-DD-AAE0-01A-11R-A41C-07 | SP112208-DO50813-T |
| TCGA-BC-A10S-01A-22R-A131-07 | SP99173-DO45225-T |
| TCGA-DD-A1EA-01A-11R-A131-07 | SP99237-DO45257-T |
| TCGA-3K-AAZ8-01A-12R-A39D-07 | SP98900-DO45093-T |
| TCGA-FV-A2QQ-01A-11R-A22L-07 | SP107136-DO48751-T |
| TCGA-WQ-AB4B-01A-11R-A41C-07 | SP112228-DO50822-T |
| TCGA-DD-AACW-01A-11R-A41C-07 | SP98959-DO45121-T |
| TCGA-DD-A39Z-01A-11R-A213-07 | SP107004-DO48677-T |
| TCGA-DD-AAE7-01A-11R-A41C-07 | SP107007-DO48679-T |
| TCGA-DD-AACH-01A-11R-A41C-07 | SP50159-DO23539-T |
| TCGA-LG-A9QD-01A-11R-A38B-07 | SP98896-DO45091-T |
| TCGA-BC-A3KF-01A-11R-A213-07 | SP112192-DO50805-T |
| TCGA-DD-A115-01A-11R-A131-07 | SP107132-DO48747-T |
| TCGA-2Y-A9H3-01A-11R-A38B-07 | SP99229-DO45253-T |
| TCGA-G3-AAUZ-01A-11R-A38B-07 | SP99221-DO45249-T |
| TCGA-BC-A217-01A-11R-A155-07 | SP107109-DO48736-T |
| TCGA-G3-AAV4-01A-11R-A38B-07 | SP107056-DO48706-T |
| TCGA-DD-AADI-01A-11R-A41C-07 | SP98967-DO45125-T |
| TCGA-FV-A3R3-01A-11R-A22L-07 | SP99109-DO45193-T |
| TCGA-CC-5261-01A-01R-A131-07 | SP50185-DO23552-T |
| TCGA-BW-A5NO-01A-11R-A27V-07 | SP99011-DO45145-T |
| TCGA-DD-A4NB-01A-12R-A266-07 | SP99271-DO45273-T |
| TCGA-2Y-A9H4-01A-11R-A38B-07 | SP107037-DO48695-T |
| TCGA-DD-AAVY-01A-11R-A41C-07 | SP99089-DO45183-T |
| TCGA-XR-A8TD-01A-12R-A39D-07 | SP107101-DO48732-T |
| TCGA-DD-AADG-01A-11R-A41C-07 | SP107091-DO48727-T |
| TCGA-DD-A4NE-01A-11R-A27V-07 | SP107123-DO48743-T |
| TCGA-2Y-A9H7-01A-11R-A39D-07 | SP99037-DO45157-T |
| TCGA-ED-A7PY-01A-11R-A33R-07 | SP107152-DO48760-T |
| TCGA-G3-A25S-01A-11R-A16W-07 | SP112288-DO50850-T |
| TCGA-DD-AAE3-01A-11R-A41C-07 | SP112304-DO50857-T |
| TCGA-YA-A8S7-01A-11R-A37K-07 | SP112300-DO50855-T |
| TCGA-CC-5260-01A-01R-A131-07 | SP107063-DO48712-T |
| TCGA-K7-A5RF-01A-11R-A28V-07 | SP112219-DO50818-T |
| TCGA-CC-A5UC-01A-11R-A28V-07 | SP99209-DO45243-T |
| TCGA-DD-A3A6-01A-11R-A22L-07 | SP50107-DO23513-T |
| TCGA-FV-A3I0-01A-11R-A22L-07 | SP99279-DO45277-T |
| TCGA-CC-A7IF-01A-11R-A33J-07 | SP112188-DO50803-T |
| TCGA-FV-A496-01A-11R-A266-07 | SP99169-DO45223-T |
| TCGA-UB-A7MD-01A-12R-A352-07 | SP99097-DO45187-T |
| TCGA-CC-A1HT-01A-11R-A131-07 | SP107010-DO48681-T |
| TCGA-WX-AA47-01A-11R-A39D-07 | SP99045-DO45161-T |
| TCGA-FV-A4ZP-01A-12R-A266-07 | SP112138-DO50778-T |
| TCGA-ED-A7XP-01A-11R-A352-07 | SP99185-DO45231-T |
| TCGA-HP-A5MZ-01A-21R-A27V-07 | SP107111-DO48737-T |
| TCGA-DD-AACE-01A-11R-A41C-07 | SP99321-DO45297-T |
| TCGA-G3-A7M8-01A-11R-A33R-07 | SP50105-DO23512-T |
| TCGA-BD-A3ER-01A-11R-A213-07 | SP99033-DO45155-T |
| TCGA-DD-A113-01A-11R-A131-07 | SP112276-DO50845-T |
| TCGA-DD-A3A8-01A-11R-A22L-07 | SP112168-DO50793-T |
| TCGA-ED-A7PX-01A-51R-A352-07 | SP99069-DO45173-T |
| TCGA-DD-AADP-01A-11R-A39D-07 | SP99105-DO45191-T |
| TCGA-2Y-A9GS-01A-12R-A38B-07 | SP112221-DO50819-T |
| TCGA-DD-A73D-01A-12R-A32O-07 | SP112273-DO50844-T |
| TCGA-KR-A7K0-01A-12R-A33R-07 | SP112182-DO50800-T |
| TCGA-WX-AA44-01A-11R-A39D-07 | SP107088-DO48725-T |
| TCGA-ED-A8O6-01A-11R-A36F-07 | SP50157-DO23538-T |
| TCGA-DD-AADU-01A-11R-A41C-07 | SP99003-DO45141-T |
| TCGA-2Y-A9H8-01A-11R-A39D-07 | SP112186-DO50802-T |
| TCGA-G3-A3CJ-01A-11R-A213-07 | SP107071-DO48716-T |
| TCGA-DD-A4NS-01A-11R-A311-07 | SP50167-DO23543-T |
| TCGA-DD-AACP-01A-11R-A41C-07 | SP98898-DO45092-T |
| TCGA-G3-A5SM-01A-12R-A28V-07 | SP112234-DO50825-T |
| TCGA-DD-AAVQ-01A-11R-A41C-07 | SP112215-DO50816-T |
| TCGA-CC-A7IJ-01A-11R-A33R-07 | SP112241-DO50829-T |
| TCGA-BC-A10U-01A-11R-A131-07 | SP112190-DO50804-T |
| TCGA-DD-AADD-01A-11R-A41C-07 | SP107076-DO48719-T |
| TCGA-ZP-A9CZ-01A-11R-A38B-07 | SP99125-DO45201-T |
| TCGA-DD-AACY-01A-11R-A41C-07 | SP99153-DO45215-T |
| TCGA-DD-A1EC-01A-21R-A131-07 | SP99305-DO45289-T |
| TCGA-UB-AA0V-01A-11R-A38B-07 | SP112213-DO50815-T |
| TCGA-DD-A1EL-01A-11R-A155-07 | SP99337-DO45305-T |
| TCGA-RC-A6M6-01A-11R-A32O-07 | SP107052-DO48704-T |
| TCGA-MI-A75I-01A-11R-A32O-07 | SP50163-DO23541-T |
| TCGA-G3-AAV6-01A-21R-A37K-07 | SP112178-DO50798-T |
| TCGA-DD-A4NG-01A-11R-A27V-07 | SP99289-DO45281-T |
| TCGA-ED-A8O5-01A-11R-A36F-07 | SP50133-DO23526-T |
| TCGA-DD-AACO-01A-11R-A41C-07 | SP191990-DO227643-T |
| TCGA-DD-AACZ-01A-11R-A41C-07 | SP98973-DO45127-T |
| TCGA-CC-5264-01A-01R-A131-07 | SP99205-DO45241-T |
| TCGA-DD-AAW3-01A-11R-A41C-07 | SP99157-DO45217-T |
| TCGA-DD-AAVX-01A-11R-A41C-07 | SP192707-DO227801-T |
| TCGA-2Y-A9HA-01A-11R-A39D-07 | SP98981-DO45131-T |
| TCGA-ED-A82E-01A-11R-A352-07 | SP50173-DO23546-T |
| TCGA-DD-AA3A-01A-11R-A37K-07 | SP98921-DO45101-T |
| TCGA-GJ-A3OU-01A-31R-A38B-07 | SP99061-DO45169-T |
| TCGA-G3-A7M9-01A-23R-A352-07 | SP192387-DO45147-T |
| TCGA-EP-A3JL-01A-11R-A213-07 | SP99287-DO45281-T |
| TCGA-DD-AAEG-01A-11R-A39D-07 | SP50171-DO23545-T |
| TCGA-UB-A7MA-01A-11R-A33R-07 | SP50131-DO23525-T |
| TCGA-XR-A8TG-01A-11R-A36F-07 | SP99093-DO45185-T |
| TCGA-DD-A3A3-01A-11R-A22L-07 | SP50151-DO23535-T |
| TCGA-DD-A1EF-01A-11R-A131-07 | SP50103-DO23511-T |
| TCGA-ED-A627-01A-12R-A311-07 | SP99161-DO45219-T |
| TCGA-DD-AACB-01A-11R-A41C-07 | SP112180-DO50799-T |
| TCGA-G3-A3CG-01A-11R-A213-07 | SP107073-DO48717-T |
| TCGA-BC-A10W-01A-11R-A131-07 | SP112224-DO50820-T |
| TCGA-G3-A25Z-01A-11R-A16W-07 | SP50155-DO23537-T |
| TCGA-DD-AADY-01A-11R-A41C-07 | SP98989-DO45135-T |
| TCGA-DD-AAEH-01A-11R-A41C-07 | SP50143-DO23531-T |
| TCGA-G3-A5SI-01A-31R-A27V-07 | SP107078-DO48720-T |
| TCGA-G3-A5SL-01A-11R-A27V-07 | SP107041-DO48697-T |
| TCGA-EP-A12J-01A-11R-A131-07 | SP99341-DO45307-T |
| TCGA-DD-AAD2-01A-11R-A41C-07 | SP107084-DO48723-T |
| TCGA-ZS-A9CF-02A-11R-A38B-07 | SP98925-DO45103-T |
| TCGA-UB-A7MB-01A-11R-A33R-07 | SP112310-DO50859-T |
| TCGA-DD-AADN-01A-11R-A41C-07 | SP98997-DO45139-T |
| TCGA-QA-A7B7-01A-11R-A32O-07 | SP99073-DO45175-T |
| TCGA-5C-A9VH-01A-11R-A37K-07 | SP98927-DO45105-T |
| TCGA-ZP-A9D4-01A-11R-A37K-07 | SP112201-DO50809-T |
| TCGA-MI-A75H-01A-11R-A32O-07 | SP99333-DO45303-T |
| TCGA-DD-AAEI-01A-11R-A41C-07 | SP112248-DO50832-T |
| TCGA-UB-A7MC-01A-11R-A33R-07 | SP107129-DO48746-T |
| TCGA-T1-A6J8-01A-11R-A32O-07 | SP99121-DO45199-T |
| TCGA-BC-4072-01B-11R-A155-07 | SP107047-DO48701-T |
| TCGA-DD-AACQ-01A-11R-A41C-07 | SP112261-DO50839-T |
| TCGA-2Y-A9GW-01A-11R-A38B-07 | SP50137-DO23528-T |
| TCGA-BC-A10X-01A-11R-A131-07 | SP99019-DO45149-T |
| TCGA-EP-A3RK-01A-11R-A22L-07 | SP107097-DO48730-T |
| TCGA-ED-A66Y-01A-11R-A311-07 | SP106993-DO48672-T |
| TCGA-DD-AADQ-01A-11R-A41C-07 | SP99257-DO45267-T |
| TCGA-2Y-A9GT-01A-11R-A38B-07 | SP106998-DO48674-T |
| TCGA-HP-A5N0-01A-11R-A28V-07 | SP50177-DO23548-T |
| TCGA-DD-A119-01A-11R-A131-07 | SP112170-DO50793-T |
| TCGA-DD-AADO-01A-11R-A41C-07 | SP99007-DO45143-T |
| TCGA-DD-A4NQ-01A-21R-A28V-07 | SP99081-DO45179-T |
| TCGA-DD-A4NL-01A-11R-A28V-07 | SP99193-DO45235-T |
| TCGA-ES-A2HS-01A-11R-A180-07 | SP112211-DO50814-T |
| TCGA-DD-AAVS-01A-11R-A41C-07 | SP50127-DO23523-T |
| TCGA-DD-A4NV-01A-11R-A311-07 | SP192714-DO50814-T |
| TCGA-BW-A5NQ-01A-11R-A27V-07 | SP99249-DO45263-T |
| TCGA-CC-A7IH-01A-11R-A33J-07 | SP50123-DO23521-T |
| TCGA-WQ-A9G7-01A-11R-A37K-07 | SP99149-DO45213-T |
| TCGA-BC-A110-01A-11R-A131-07 | SP112205-DO50811-T |
| TCGA-RC-A7SB-01A-21R-A352-07 | SP107121-DO48742-T |
| TCGA-BC-A10T-01A-11R-A131-07 | SP192726-DO50859-T |
| TCGA-MR-A8JO-01A-12R-A36F-07 | SP107150-DO48759-T |
| TCGA-2Y-A9GX-01A-11R-A38B-07 | SP99177-DO45227-T |
| TCGA-K7-A5RG-01A-11R-A28V-07 | SP99301-DO45287-T |
| TCGA-UB-AA0U-01A-11R-A38B-07 | SP99181-DO45229-T |
| TCGA-DD-AAD3-01A-11R-A41C-07 | SP98953-DO45117-T |
| TCGA-DD-A1ED-01A-11R-A155-07 | SP99049-DO45163-T |
| TCGA-DD-AAVZ-01A-11R-A41C-07 | SP50145-DO23532-T |
| TCGA-CC-A123-01A-11R-A131-07 | SP107155-DO48761-T |
| TCGA-DD-A11B-01A-11R-A131-07 | SP107032-DO48693-T |
| TCGA-G3-A25Y-01A-11R-A16W-07 | SP99133-DO45205-T |
| TCGA-NI-A4U2-01A-11R-A28V-07 | SP112160-DO50789-T |
| TCGA-EP-A2KC-01A-11R-A213-07 | SP99201-DO45239-T |
| TCGA-RC-A6M5-01A-11R-A32O-07 | SP107068-DO48715-T |
| TCGA-2Y-A9GU-01A-11R-A38B-07 | SP99101-DO45189-T |
| TCGA-G3-AAV2-01A-11R-A37K-07 | SP99329-DO45301-T |
| TCGA-DD-AAEA-01A-11R-A41C-07 | SP99233-DO45255-T |
| TCGA-DD-A4ND-01A-11R-A266-07 | SP107012-DO48682-T |
| TCGA-DD-A4NO-01A-11R-A28V-07 | SP112130-DO50774-T |
| TCGA-RC-A7SK-01A-11R-A352-07 | SP99269-DO45273-T |
| TCGA-CC-A3MB-01A-11R-A213-07 | SP99085-DO45181-T |
| TCGA-DD-A4NA-01A-11R-A266-07 | SP50117-DO23518-T |
| TCGA-DD-AAE2-01A-11R-A41C-07 | SP112196-DO50807-T |
| TCGA-RG-A7D4-01A-12R-A33R-07 | SP98933-DO45107-T |
| TCGA-LG-A9QC-01A-11R-A37K-07 | SP50135-DO23527-T |
| TCGA-DD-A3A9-01A-11R-A266-07 | SP112147-DO50783-T |
| TCGA-G3-A25V-01A-11R-A16W-07 | SP50101-DO23510-T |
| TCGA-ZP-A9D1-01A-11R-A38B-07 | SP99217-DO45247-T |
| TCGA-BC-A216-01A-11R-A155-07 | SP107080-DO48721-T |
| TCGA-ZP-A9D2-01A-11R-A38B-07 | SP98937-DO45109-T |
| TCGA-DD-A1EK-01A-11R-A213-07 | SP50111-DO23515-T |
| TCGA-RC-A6M4-01A-11R-A32O-07 | SP98955-DO45119-T |
| TCGA-DD-A1EH-01A-11R-A131-07 | SP112217-DO50817-T |
| TCGA-ED-A7PZ-01A-11R-A33R-07 | SP50161-DO23540-T |
| TCGA-CC-A9FS-01A-11R-A37K-07 | SP98965-DO45123-T |
| TCGA-CC-A3MA-01A-11R-A213-07 | SP99297-DO45285-T |
| TCGA-DD-A4NH-01A-11R-A27V-07 | SP112152-DO50785-T |
| TCGA-FV-A2QR-01A-11R-A213-07 | SP98991-DO45137-T |
| TCGA-2Y-A9H5-01A-11R-A38B-07 | SP50175-DO23547-T |
| TCGA-5C-A9VG-01A-11R-A37K-07 | SP99197-DO45237-T |
| TCGA-PD-A5DF-01A-11R-A27V-07 | SP99057-DO45167-T |
| TCGA-DD-AAD6-01A-11R-A41C-07 | SP99001-DO45141-T |
| TCGA-DD-A73F-01A-11R-A32O-07 | SP99283-DO45279-T |
| TCGA-DD-A1EI-01A-11R-A131-07 | SP99245-DO45261-T |
| TCGA-ZS-A9CF-01A-11R-A38B-07 | SP99225-DO45251-T |
| TCGA-RC-A7SH-01A-11R-A38B-07 | SP50115-DO23517-T |
| TCGA-ZS-A9CE-01A-11R-A37K-07 | SP99241-DO45259-T |
| TCGA-DD-A4NJ-01A-11R-A27V-07 | SP192418-DO45173-T |
| TCGA-DD-AAE1-01A-11R-A41C-07 | SP50129-DO23524-T |
| TCGA-DD-A39X-01A-11R-A213-07 | SP98949-DO45115-T |
| TCGA-DD-AACG-01A-11R-A41C-07 | SP112165-DO50791-T |
| TCGA-DD-A4NN-01A-11R-A28V-07 | SP50181-DO23550-T |
| TCGA-K7-A6G5-01A-11R-A311-07 | SP98902-DO45094-T |
| TCGA-2Y-A9H1-01A-11R-A38B-07 | SP99253-DO45265-T |
| TCGA-MI-A75C-01A-11R-A32O-07 | SP107146-DO48757-T |
| TCGA-ED-A66X-01A-11R-A311-07 | SP98907-DO45096-T |
| TCGA-DD-AAW1-01A-11R-A41C-07 | SP50119-DO23519-T |
| TCGA-DD-AADB-01A-11R-A41C-07 | SP50169-DO23544-T |
| TCGA-G3-AAV1-01A-11R-A38B-07 | SP107044-DO48700-T |
| TCGA-BC-A10R-01A-11R-A131-07 | SP99145-DO45211-T |
| TCGA-G3-A25X-01A-11R-A16W-07 | SP112194-DO50806-T |
| TCGA-DD-A3A4-01A-11R-A22L-07 | SP98945-DO45113-T |
| TCGA-BC-A10Z-01A-11R-A131-07 | SP112156-DO50787-T |
| TCGA-DD-A4NR-01A-11R-A311-07 | SP50183-DO23551-T |
| TCGA-CC-A5UE-01A-11R-A28V-07 | SP50147-DO23533-T |
| TCGA-CC-A9FW-01A-11R-A37K-07 | SP99265-DO45139-T |
| TCGA-XR-A8TC-01A-11R-A36F-07 | SP112210-DO50814-T |
| TCGA-BC-A69I-01A-11R-A311-07 | SP99029-DO45153-T |
| TCGA-DD-AACX-01A-11R-A41C-07 | SP99041-DO45159-T |
| TCGA-DD-AAW2-01A-11R-A41C-07 | SP99293-DO45283-T |
|  | SP50179-DO23549-T |
|  | SP99129-DO45203-T |
|  | SP98915-DO45099-T |
|  | SP112198-DO50808-T |
|  | SP107094-DO48728-T |
|  | SP112265-DO50840-T |
|  | SP99117-DO45197-T |
|  | SP99261-DO45269-T |
|  | SP107119-DO48741-T |
|  | SP98941-DO45111-T |
|  | SP98904-DO45095-T |
|  | SP50141-DO23530-T |
|  | SP98909-DO45096-T |
|  | SP50165-DO23542-T |
|  | SP99141-DO45209-T |
|  | SP99165-DO45221-T |
|  | SP50097-DO23508-T |
|  | SP112133-DO50776-T |
|  | SP98975-DO45129-T |
|  | SP50113-DO23516-T |
|  | SP50099-DO23509-T |
