# Supplementary material for: m6A RNA Methylation Regulators Elicit Malignant Progression and Predict Clinical Outcome in Hepatocellular Carcinoma
Source: Dis Markers. 2021 Jun 4;2021:8859590. doi: 10.1155/2021/8859590 (PMC8218914; doi:10.1155/2021/8859590)
Supplement: Supplementary 2 — Table S1: accession ID. [file 8859590.f2.docx]

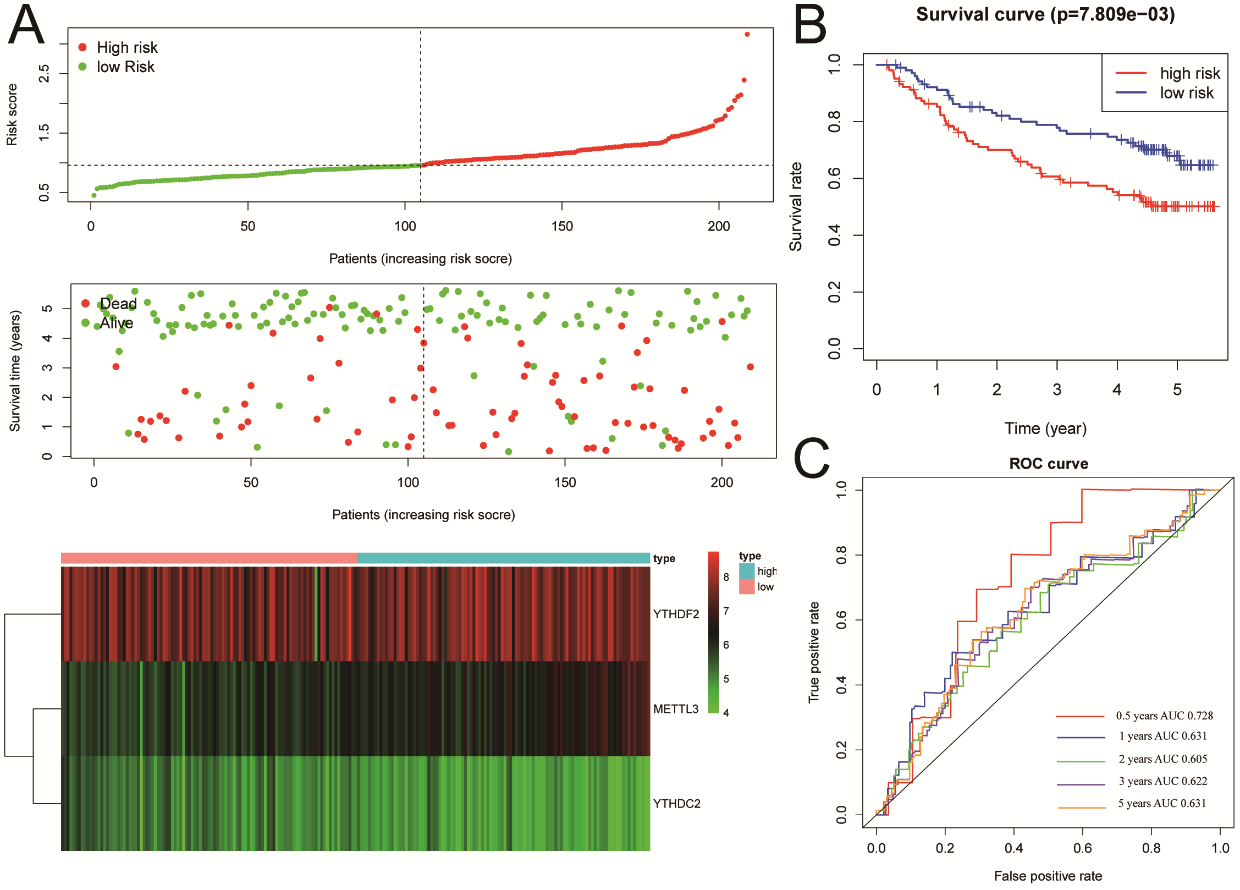


Figure S1 verification of prognostic signature in GSE14520 cohort. (A) The distribution of risk scores (upper), survival time (middle) and gene expression levels (below) (B) The Kaplan-Meier survival curves between high- and low-risk groups. (C) ROC curves and AUC values of the signature.
